# Profiles of Secondary Metabolites (Phenolic Acids, Carotenoids, Anthocyanins, and Galantamine) and Primary Metabolites (Carbohydrates, Amino Acids, and Organic Acids) during Flower Development in *Lycoris radiata*

**DOI:** 10.3390/biom11020248

**Published:** 2021-02-09

**Authors:** Chang Ha Park, Hyeon Ji Yeo, Ye Jin Kim, Bao Van Nguyen, Ye Eun Park, Ramaraj Sathasivam, Jae Kwang Kim, Sang Un Park

**Affiliations:** 1 Department of Crop Science, Chungnam National University, 99 Daehak-ro, Yuseong-gu, Daejeon 34134, Korea; parkch804@gmail.com (C.H.P.); guswl7627@gmail.com (H.J.Y.); yeney1996@cnu.ac.kr (Y.E.P.); ramarajbiotech@gmail.com (R.S.); 2 Division of Life Sciences, College of Life Sciences and Bioengineering, Incheon National University, Yeonsugu, Incheon 22012, Korea; 201721047@inu.ac.kr; 3 Department of Smart Agriculture Systems, Chungnam National University, 99 Daehak-ro, Yuseong-gu, Daejeon 34134, Korea; nguyenvanbao@tuaf.edu.vn

**Keywords:** *Lycoris radiata*, flower development, metabolic profiling, primary metabolites, secondary metabolites

## Abstract

This study aimed to elucidate the variations in primary and secondary metabolites during *Lycoris* *radiata* flower development using high performance liquid chromatography (HPLC) and gas chromatography time-of-flight mass spectrometry (GC-TOFMS). The result showed that seven carotenoids, seven phenolic acids, three anthocyanins, and galantamine were identified in the *L*. *radiata* flowers. Most secondary metabolite levels gradually decreased according to the flower developmental stages. A total of 51 metabolites, including amines, sugars, sugar intermediates, sugar alcohols, amino acids, organic acids, phenolic acids, and tricarboxylic acid (TCA) cycle intermediates, were identified and quantified using GC-TOFMS. Among the hydrophilic compounds, most amino acids increased during flower development; in contrast, TCA cycle intermediates and sugars decreased. In particular, glutamine, asparagine, glutamic acid, and aspartic acid, which represent the main inter- and intracellular nitrogen carriers, were positively correlated with the other amino acids and were negatively correlated with the TCA cycle intermediates. Furthermore, quantitation data of the 51 hydrophilic compounds were subjected to partial least-squares discriminant analyses (PLS-DA) to assess significant differences in the metabolites of *L*. *radiata* flowers from stages 1 to 4. Therefore, this study will serve as the foundation for a biochemical approach to understand both primary and secondary metabolism in *L*. *radiata* flower development.

## 1. Introduction

The *Lycoris* species, belonging to the Amaryllidaceae family, is native to eastern Asian countries like Korea, China, and Japan, and mainly inhabit moist, warm-temperate woodlands [1]. Among them, *Lycoris radiata* is considered medicinally and ornamentally important, as the flowers have been used for decoration and the bulbs are known to produce alkaloids with various medicinal properties [2]. In particular, galantamine is a tertiary alkaloid isolated from the bulbs or flowers of Amaryllidaceae plants and is used for the treatment of mild to moderate Alzheimer disease. In addition, alkaloids identified in this plant species have beneficial effects such as anti-inflammatory and cytotoxic activities [3], antimalarial effects [4], and an ability to inhibit anti-insulin antibodies in non-obese diabetic patients [5].

Flower color is an essential trait in ornamental plants. Flower coloration is determined by the grade of accumulation of pigments, including anthocyanins, betalains, and carotenoids. Aside from increasing commercial value, flower colors contribute to attracting pollinators such as animals and insects, defend against UV radiation, and act as key signals between microbes and plants [6,7,8].

Carotenoids are members of the terpenoid class and are involved in the color development of yellow, red, or orange coloration in plants [9,10]. In non-green tissues such as seeds, fruits, and flowers, carotenoids are produced in the chromoplasts, causing the tissues to exhibit vibrant colors; however, in green tissues, these pigments play significant roles in photosynthesis including photosystem assembly, light harvesting, and photoprotective functions [10,11,12]. Furthermore, the carotenoid content in non-green tissues varies significantly according to the plant species, while green tissues of most plants exhibit similar carotenoid profiles [12,13,14].

Anthocyanins, belonging to the flavonoid class, are natural pigments imparting a broad range of colors such as purple, blue, orange, and red to flowers. There are six common anthocyanidins (i.e., cyanidin, delphinidin, malvidin, pelargonidin, peonidin, and petunidin) [8,15,16,17], and cyanidin is a dominant anthocyanin aglycone present in plants that is responsible for red-purple coloration [18]. Chun et al. [19] previously reported that cyanidin is a major anthocyanidin present in *L*. *radiata* flowers by identifying the presence of cyanidin 3-diglucoside, cyanidin 3-sambubioside, and cyanidin 3-glucoside.

**Figure 1:**
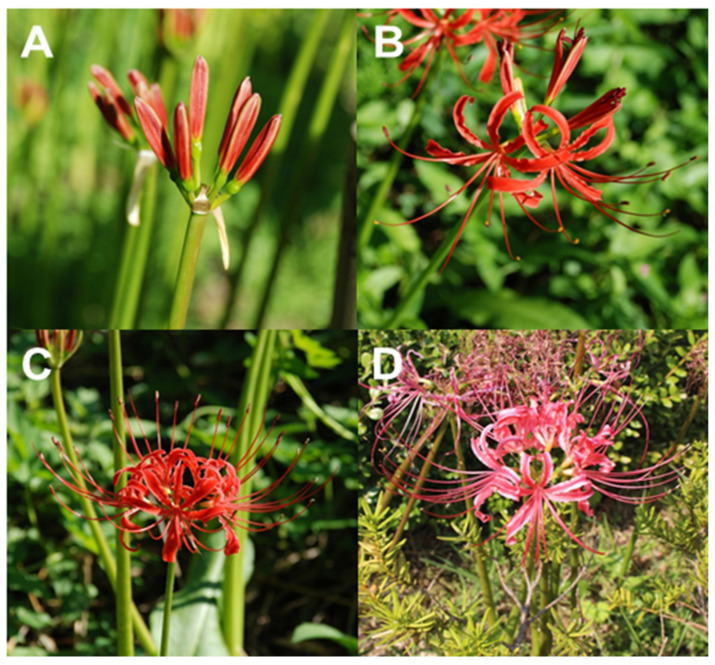
Four stages of *Lycoris radiata* flower development. (**A**) Floral bud (stage 1); (**B**) partially opened flower (stage 2); (**C**) fully opened flower (stage 3); and (**D**) senescent flower (stage 4).

A model for petal development and senescence was suggested by Ma et al. [20], wherein they divided petal development into three phases, namely, the cell division phase, cell expansion phase, and cell shrink/death phase. Briefly, petal senescence may be initiated when petal cells cease dividing and enter the cell expansion phase, and such senescence may be synergistically regulated by various factors such as epigenetic modifiers, hormones, kinases, other enzymes, micro RNAs (miRNAs), sugar levels, and transcription factors [20]. Among these factors, endogenous sugar levels were altered during flower opening and senescence, and the sucrose supply promoted flower opening and delayed senescence by changing hormonal balance [21]. Therefore, metabolite profiling may offer beneficial information that would extend and complement previous studies. This study aimed to provide valuable information on metabolite changes according to the four different floral development stages of *Lycoris radiata* (Figure 1) and to determine the possible relationships between diverse metabolites including primary (amine, sugars, sugar intermediates, sugar alcohols, amino acids, organic acids, and tricarboxylic acid (TCA) cycle intermediates) and secondary metabolites (phenolics, carotenoids, and alkaloids).

## 2. Materials and Methods

### 2.1. Plant Materials

Three biological replicates of *L*. *radiata* flowers at stage 1 (floral bud), stage 2 (partially opened flower), stage 3 (fully opened flower), and stage 4 (senescent flower) were harvested from the experimental field of Chungnam National University in October 2019 and were snap-frozen using liquid nitrogen (−196 °C). Each biological replicate contained a pool of 10 flowers harvested at the four different developmental stages. The harvested flowers were then lyophilized at −80 °C for 72 h, after which a mortar and pestle were used to grind the flower samples for further analyses.

### 2.2. Carotenoid HPLC Analysis

Carotenoid HPLC analysis was performed using a previously reported method [9]. Dried powders (0.02 g) of *L*. *radiata* flowers from different developmental stages were extracted using 0.1% (*w*/*v*) ascorbic acid in ethanol (3 mL) and vortexed for 20 s. Extracts were placed in a water bath set to 85 °C for 5 min. For saponification, potassium hydroxide (120 μL, 80% *w*/*v*) was added to the extracts and the extracts were placed in a water bath at 85 °C for 10 min. Afterwards, extracts were immediately transferred on ice, and cold distilled water (1.5 mL) was added to each tube. Trans-β-apo-8′-carotenal (0.2 mL, 25 μg/mL) was used as an internal standard. Hexane (1.5 mL) was used to separate layers, and the upper hexane layer of each sample was transferred into new tubes. This step was repeated two more times. The collected supernatants were dried using nitrogen gas and dissolved in 0:50 (*v*/*v*) dichloromethane/methanol (0.25 mL). The analytical equipment and conditions used for HPLC analysis were carried out as described by Park et al. [9]. Carotenoids were identified using our previous library and, through the combined use of retention time and co-elution with trans-β-apo-8′-carotenal, were quantitated with reference to the corresponding calibration curves. The linear equations were y = 0.1847x + 0.1214, y = 0.4712x − 0.0284, y = 0.3329x + 0.0338, y = 0.3361x − 0.0220, y = 0.5479x − 0.0805, y = 0.2154x + 0.1814, and y = 0.4284x + 0.0339 for lutein, zeaxanthin, β-cryptoxanthin, 13*Z*-β-carotene, α-carotene, β-carotene, and 9*Z*-β-carotene, respectively.

### 2.3. Phenolic Acid HPLC Analysis

Phenolic acid analysis was performed using a previously reported method [22]. Dried powders (0.1 g) of *L*. *radiata* flowers from the different developmental stages were extracted using 80% methanol (1 mL) and sonicated for 30 min at 25 °C. After centrifugation at 15,000× *g* for 15 min, supernatants were transferred to new tubes. This extraction procedure was repeated two times using the sample residues. An HPLC analysis system, consisting of an OptimaPak C18 column (250 × 4.6 mm, 5 μm; RStech Co., Daejeon, Korea), a NS-4000 HPLC system, a NS-6000 auto-sampler (Futecs Co., Daejeon, Korea), a degasser, and a UV-Vis detector, was used to separate and quantify the polyphenol compounds in the sample extracts. The analytical conditions for the phenolic acid analysis were carried out based on our previous study [22]. Phenolic acids were identified by comparison with the retention time of gallic acid, 4-hydroxybenzoic acid, chlorogenic acid, caffeic acid, and *p*-coumaric acid standard chemical (Sigma-Aldrich Korea, Yongin, Korea) using the spiking test and were quantified with the corresponding calibration curves. The linear equations were y = 32.896x − 26.174, y = 16.421x + 100.8, y = 17.795x − 70.351, y = 39.983x − 65.708, and y = 60.933x + 266.03 for gallic acid, 4-hydroxybenzoic acid, chlorogenic acid, caffeic acid, and p-coumaric acid, respectively.

### 2.4. Galantamine HPLC Analysis

Galantamine HPLC analysis was performed using a previously reported method with slight modification [2]. Powders (0.1 g) of *L*. *radiata* flowers at the different developmental stages were extracted using 80% methanol (1 mL) and then sonicated for 30 min at 25 °C. After centrifugation at 15,000× *g* for 15 min, supernatants were then transferred into new tubes. This procedure was repeated twice using the sample residues. The collected solutions were dried using nitrogen gas and dissolved in methanol. The OptimaPak C18 column, a NS-4000 HPLC system, a NS-6000 auto-sampler, a degasser, and a UV-Vis detector were used for the isolation of galantamine. The analytical conditions and identification and quantification of the galantamine content were estimated based on our previous study [2]. The linear equation was y = 12.770x − 12.500 for galantamine.

### 2.5. Anthocyanin HPLC Analysis

Anthocyanin HPLC analysis was performed according to the procedure described in our previous study, which reported the identification of cyanidin 3-diglucoside (C3DG), cyanidin 3-sambubioside (C3S), and cyanindin-3-glucoside (C3G) with their mass spectra *m*/*z* 318 [M+H]+, 287 MS^n^ for C3DG, *m*/*z* 581 [M+H]+, 287 MS^n^ for C3S, and *m*/*z* 449 [M+H]+, 287 MS^n^ for C3G in *L*. *radiata* flowers [19]. Powders (0.1 g) of *L*. *radiata* flowers at different developmental stages were extracted using 1.5 mL water/methanoic acid (95:5, *v*/*v*) and then mildly sonicated for 20 min at 25 °C. After centrifugation at 8000 rpm for 30 min, supernatants were filtered through a Polytetrafluorethylene (PTFE) hydrophilic syringe. The HPLC system (Agilent Technologies, Palo Alto, CA, USA) including a Synergy 4-μm Polar-RP 80A column (250 × 4.6 mm id; Phenomenex, Torrance, CA, USA) with a Security Guard Cartridge Kit (AQ C18, 4 × 3 mm id; Phenomenex, Torrance, CA, USA) was for the isolation of anthocyanins. The analytical conditions and identification and quantification of the anthocyanin content was carried out based on our previous study [19]. The linear equations were y = 6.042x − 4.204 for C3G and the concentrations of C3DG and C3S were calculated as equivalents of C3G.

### 2.6. QRT-PCR Analysis

The qRT-PCR was carried out using a previously reported method [23]. Total RNA from *L*. *radiata* flowers from the different developmental stages was extracted using Plant RNA Mini Kit (Geneaid, Sijhih, Taiwan) and then complementary DNA (cDNA) synthesis was performed using The ReverTraAce^®^ Kit (TOYOBO, Osaka, Japan). The gene specific primers from the previous study were used in this study. The analytical equipment and conditions used for GC-TOFMS analysis were carried out as described by Park et al. [23]. The expression of phenylpropanoid and galantamine biosynthetic genes was calculated using the 2^−ΔΔ*Ct*^ method.

### 2.7. GC-TOFMS Analysis for Carbohydrate, Amino Acid, Organic Acid, and Phenolic Acid

The extraction and analysis were performed using our previously reported methods [24]. Dried powders (0.01 g) of *L*. *radiata* flowers at different developmental stages were extracted using 1 mL of a water/chloroform/methanol (1:1:2.5 *v/v*/*v*) mixture, to which 60 µL of adonitol (0.2 g L^−1^) were added as an internal standard. Extracts were mixed in an Eppendorf ThermoMixer^®^ Comfort at a shaking frequency of 1300 rpm and at 37 °C for 30 min, followed by centrifugation at 15,000× *g* for 10 min. The polar phase (800 µL) was transferred into a new tube, and 400 µL of distilled water was then added. This mixture was then centrifuged at 15,000× *g* for 5 min, and the upper layer (900 µL) was transferred into a new tube. Resulting aliquots were freeze-dried in a centrifugal concentrator (CC-105) for 16 h, followed by the addition of 80 μL methoxyamine hydrochloride. Afterwards, incubation at 30 °C for 100 min with a shaking frequency of 1300 rpm using an Eppendorf ThermoMixer^®^ Comfort was performed. Next, 80 μL of N-methyl-N-trimethylsilyl trifluoroacetamide was added to the mixture, which was then subsequently incubated at 37 °C and 1300 rpm for 30 min, using an Eppendorf ThermoMixer^®^ Comfort for derivatization. Analytical equipment and conditions used for GC-TOFMS analysis were based on Park et al. [24]. Quantification was performed using selected ions, and the Chroma-TOF software was used to locate the peaks.

### 2.8. Statistical Analysis

Analysis of variance (ANOVA) with Duncan’s multiple range test (DMRT) set to *p* < 0.05 was used for data analysis. This was done using SAS software version 9.2 (SAS Institute Inc., Cary, NC, USA). Principal component analysis (PCA), partial least-squares discriminant analysis (PLS-DA), and hierarchical cluster analysis (HCA) with Pearson correlation for the metabolites detected in this study were performed using the MetaboAnalyst 3.0 (http://www.metaboanalyst.ca/) with auto-scaling (mean-centered and divided by the standard deviation of each variable).

## 3. Results and Discussion

### 3.1. Carotenoids in Flowers at Different Flower Developmental Stages

Analysis of flowers at the three different developmental stages revealed seven types of carotenoids: lutein, zeaxanthin, β-cryptoxanthin, 13*Z*-β-carotene, α-carotene, β-carotene, and 9*Z*-β-carotene (Table 1 and Figure S1). Among the seven carotenoids, the levels of six carotenoids (zeaxanthin, β-cryptoxanthin, 13*Z*-β-carotene, α-carotene, β-carotene, and 9*Z*-β-carotene) were decreased. In particular, the highest levels of β-cryptoxanthin, *α*-carotene, β-carotene, and 9*Z*-β-carotene were recorded in stage 1, and the highest levels of zeaxanthin and 13*Z*-β-carotene were observed in stage 2. In contrast, an increasing pattern was observed for lutein from stage 1 to stage 4.

### 3.2. Galantamine in Flowers at the Different Flower Developmental Stages

HPLC analysis confirmed the presence of galantamine in flower samples at the different flower developmental stages (Table 2 and Figure S2), including stage 1 (2.71 ± 0.13 mg/g Dry Weight (DW)), stage 2 (2.39 ± 0.17 mg/g DW), stage 3 (1.65 ± 0.01 mg/g DW), and stage 4 (1.89 ± 0.08 mg/g DW). The galantamine content gradually decreased according to the developmental flower stage. Galantamine accumulated at the highest level in stage 1, and it was 1.13, 1.65, and 1.89 times higher than the galantamine contents of stages 2, 3, and 4, respectively. These results were correlated with the expression levels of *LrN4OMT* and *LrCYP96T*, which are important for the galantamine biosynthesis (Figure S3).

### 3.3. Phenolic Acids and Anthocyanins in Flowers at Different Flower Developmental Stages

HPLC analysis of flowers at the three different developmental stages revealed five types of phenolic acid: gallic acid, 4-hydroxybenzoic acid, chlorogenic acid, caffeic acid, and *p*-coumaric acid, whereas GC-TOFMS-based analysis revealed two types of phenolic acid: ferulic acid and sinapinic acid (Table 3 and Figure S4). Most levels of phenolic acids gradually decreased during flower development except for chlorogenic acid. The highest levels of gallic acid (74.51 ± 5.50 µg/g DW), caffeic acid (143.96 ± 17.31 µg/g DW), *p*-coumaric acid (26.24 ± 2.36 µg/g DW), and ferulic acid (0.06 ± 0.01 intensity/g) were observed at stage 1, and the highest levels of 4-hydroxybenzoic acid and sinapinic acid were recorded at stages 1 and 2. In contrast, the level of chlorogenic acid increased from stage 1 to stage 3, and then decreased from stage 3 to stage 4.

Furthermore, HPLC analysis confirmed the presence of cyanidin 3-diglucoside, cyanidin 3-sambubioside, and cyanindin-3-glucoside in flower samples at the different flower developmental stages (Table 4 and Figure S5). The three anthocyanins increased during the flower development stages and then decreased in the senescence stage. Among the individual anthocyanins, cyanidin 3-diglucoside, cyanidin 3-sambubioside, and cyanindin-3-glucoside increased from stage 1 to stage 3 and decreased from stage 3 to stage 4. In particular, cyanidin 3-sambubioside was the major anthocyanin in *L*. *radiata* flowers. The highest levels of the three anthocyanins were observed in stages 2 and 3. Furthermore, these findings were in agreement with the expression patterns of phenylpropanoid biosynthesis genes studied in this study (Figure S3).

Decreasing patterns of phenolic acid, anthocyanin, carotenoid, and galantamine contents were observed according to flower developmental stages of *L*. *radiata*. Therefore, this study suggested the reduction in the accumulation of various secondary metabolites during flower development. Previous studies supported our results. For example, carotenoid levels decreased during the four stages of development of *Ipomoea obscura*, which has pale yellow petals, and *Ipomoea nil*, which has white petals, but the levels increased during the four flower stages of *Ipomoea* sp., which has yellow petals [25]. Yamagishi et al. [26] reported that *Lilium* ‘Montreux’, which has pink tepals, revealed decreasing patterns of total carotenoid contents in the four stages of tepals; however, the other cultivar, *Lilium* ‘Connecticut King’, which has yellow tepals, showed increasing patterns of carotenoid contents, which is consistent with our study. Among six rose cultivars (*Rosa* ‘Yellow Island’, *Rosa* ‘Chacok’, *Rosa* ‘Garden City’, *Rosa* ‘Yunzheng Xiawei’, *Rosa* ‘Wangri qinghuai’, and *Rosa* ‘Pink Fan’), the greatest levels of carotenoids, flavonols, and anthocyanins increased and then decreased according to the seven different developmental stages [27]. Phenolic acids, including gallic acid, caffeic acid, and *p*-coumaric acid, as well as anthocyanins, decreased according to the flower stages of rose cultivar ‘KORcrisett’ [28], and a decreasing pattern of total phenolic content was observed in the petals of eight cultivars of *Rosa* × *hybrida* (‘NOAschnee’, ‘KOReb’, ‘Sea Foam’, ‘The Fairy’, ‘KORverlandus’, ‘POUleas’, ‘MORedfar’, and ‘Gärtnerfreude’) during the four flower developmental stages [29]. Nisar et al. [30] reported that the total phenolic content decreased after flower opening of *Nicotiana plumbaginifolia*. Not only did galantamine content increase at the blooming stage, it also decreased after blooming in *Narcissus poeticus* [31]; moreover, galantamine content was observed to increase and then decrease based on the flower development of *Narcissus confusus* [32].

### 3.4. Metabolic Profiling and Multivariate Analysis

**Figure 2:**
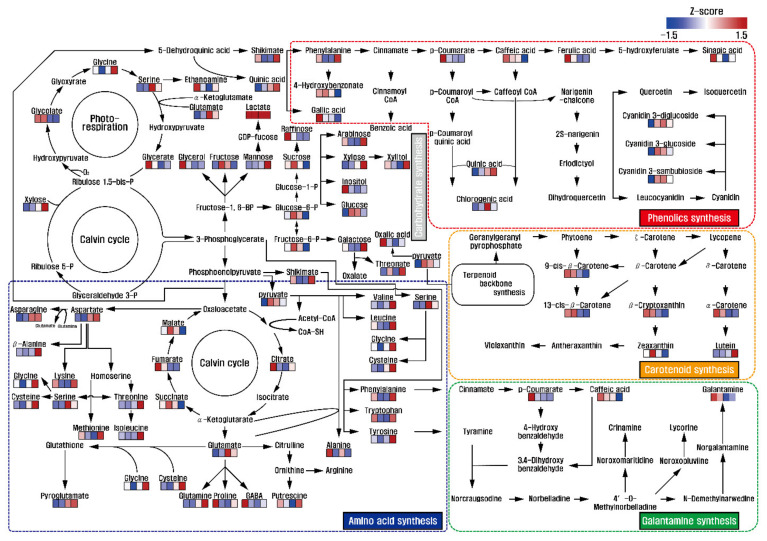
Overview of metabolic changes in different developmental stages of *L*. *radiata* flowers. The scale bar indicates the z-score transformed average values of metabolites, and the colored squares (blue-to-red) represent relative metabolite abundance in the different flower developmental stages.

Identification and quantification of low-molecular-weight molecules from *L*. *radiata* flowers at the different developmental stages were performed. A total of 51 metabolites (one amine, eight sugars, two sugar intermediates, three sugar alcohols, 22 amino acids, nine organic acids, two phenolic acids, and four TCA cycle intermediates) were detected in the flowers (Figures S6 and S7). Among the TCA cycle intermediates, the levels of succinic acid, fumaric acid, and malic acid showed decreasing patterns during flower development. However, increasing patterns of amino acids including glutamine, glutamic acid, asparagine, aspartic acid, pyroglutamic acid, threonine, valine, tyrosine, isoleucine, glycine, cysteine, serine, and beta-alanine were observed. Similarly, levels of lysine, phenylalanine, tryptophan, methionine, leucine, and putrescine decreased from stage 1 to stage 2 and then increased from stage 3 to stage 4. In contrast, decreasing patterns of 4-aminobutyric acid and alanine were observed during development. Therefore, most amino acid levels were higher in stage 1. In sugars, sugar alcohols, and sugar intermediates, the levels of fructose, glucose, sucrose, fructose-6-phosphate, and glucose-6-phosphate slightly increased from stage 1 to stage 2 and then gradually decreased from stage 2 to stage 3. Similarly, decreasing patterns of raffinose, glycerol, and inositol were observed during flower development. On the other hand, xylose and mannose levels gradually increased (Figure 2).

Thus, this study indicated that total sugars, consisting mainly of sugars, sugar alcohols, and sugar intermediates in this study, increased and then decreased during flower development. These findings were consistent with previous studies reporting a decrease in sugars in different developmental floral stages in many plant species. For example, total sugars (reducing and non-reducing sugars) and starch contents decreased in the different floral development stages of *Lilium pumilum* (belonging to Liliaceae), and the concentration of soluble sugar and starch declined, while celluloses, hemicelluloses, and lignin gradually increased in the four different flower developmental stages of *Gentiana macrophylla* [33]. Furthermore, total sugars, including reducing and non-reducing sugars, gradually increased and then decreased during floral development and senescence of *Narcissus tazetta* (*N*. *tazetta*) [34], *Consolida ajacis* (*C*. *ajacis*) [35], and *Ranunculus asiaticus* L. (*R*. *asiaticus* L.) [36], consistent with the present study that reported a decrease after an increase in total sugar content. Furthermore, correlation between total sugars, consisting mainly of sugars, sugar alcohols, and sugar intermediates, and secondary metabolites, including carotenoids, phenolic acids, and galantamine, gradually decreased in *L*. *radiata* flowers from stage 1 to stage 4, reflecting energy demand to support secondary metabolism. In particular, decreasing patterns of sucrose, fructose, and glucose were observed. The production of galantamine, carotenoids, and phenolic acids was likely to be positively correlated with sucrose concentrations. For instance, higher concentrations of galantamine and sucrose were observed in the bulbs of *L*. *radiata*, followed by roots and leaves, and the optimum sucrose concentration improved the production of galantamine in the shoot cultures of *Galanthus elwesii* [37], *Narcissus pseudonarcissus* [37], and *Leucojum aestivum* [37,38], as well as in shoot-clump cultures of *N*. *confusus* (belonging to Amaryllidaceae) [39]. Furthermore, a positive relationship between sucrose and carotenoids including lutein, lycopene, and beta-carotene was observed during watermelon fruit development [40]. Additionally, sucrose deficiency induced the delay of lycopene accumulation in fruit pericarp discs of *Solanum lycopersicum* [41], but the exogenous addition of sucrose increased carotenoid content in the juice sacs of *Satsuma mandarin*, *Valencia orange*, and *Lisbon lemon* [42], as well as in cell cultures of *Daucus carot* [43]. Previous studies also reported that sucrose affects biosynthesis of phenolic compounds. Calcium signaling, which is initiated by an exogenous supply of sucrose, leads to sucrose uptake through the transcriptional regulation of sucrose transporter 1 (SUC1), and the improved endogenous sugar abundances induced anthocyanin accumulation, activating regulatory genes involved in anthocyanin biosynthesis [44]. Zakhleniuk et al. [45] reported that the *pho3* mutant, having a SUC2 deficiency, showed large abundance of soluble sugars, such as starch, sucrose, fructose, and glucose, and improved production of anthocyanins in *Arabidopsis* plants. Additionally, the strong positive correlation between sucrose and flavones (baicalein, baicalin, and wogonin) in *PAP1*-overexpressing hairy roots of *Scutellaria baicalensis* [46] as well as positive correlations between sucrose and phytochemicals (anthocyanin and carotenoid) in the leaves and fruits of the diploid and tetraploid cultivar *Morus alba* [47] were reported.

**Figure 3:**
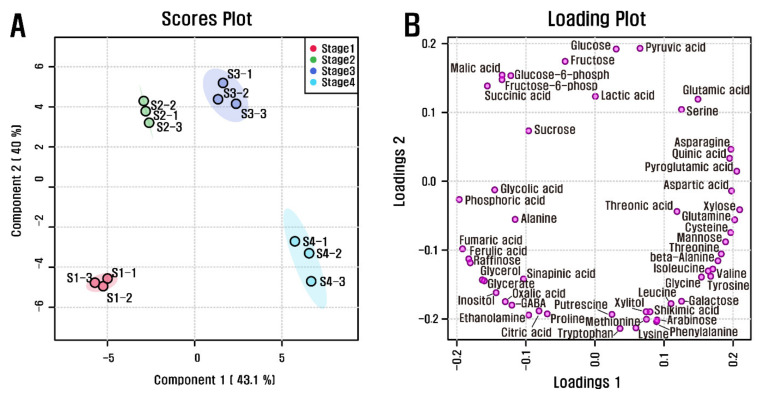
(**A**) Scores and (**B**) loading plots of PLS-DA model obtained from 51 metabolites from *L*. *radiata* at the different flower developmental stages using GC-TOFMS.

Quantitative data for the 51 polar metabolites were subjected to PCA to investigate variations in metabolic profiles from stages 1 to 4 of the *L*. *radiata* flowers (Figure S8). The two highest-ranking components based on PCA accounted for 48.3% and 35.3% of the total variance. In particular, the first component resolved the measured metabolite profiles of stage 4 from stages 1, 2, and 3. This separation was mainly attributable to amino acids, carbohydrates, and organic acids. The metabolites were tyrosine, beta-alanine, valine, threonine, and isoleucine, which had eigenvector values of 0.20413, 0.20128, 0.20097, 0.19926, and 0.19852, respectively, and succinic acid, malic acid, fructose-6-phosphate, glucose-6-phosphate, phosphoric acid, and fructose, which had eigenvector values of −0.19429, −0.18551, −0.18206, −0.17551, −0.1358, and −0.12515, respectively. This supported the idea that most levels of amino acids were higher in stage 4. To maximize separation from stage 1 to stage 4 of the *L*. *radiata* flowers, partial least-squares discriminant analysis (PLS-DA) was performed (Figure 3). The coefficient of determination (R^2^) and the cross-validated coefficient of determination (Q^2^) values of the PLS-DA model (0.97886 and 0.92695, respectively) revealed that the model showed goodness of fit and had reliable predictive capability. Among the flowers at different developmental stages, PLS-DA with the first two components, accounting for 43.1% and 40% of the total variance, indicated clear separation. Variable importance in projection (VIP) is a metric used to assess the contribution of variables in a data set. Generally, a value >1.0 is the recommended VIP criterion for identifying and validating the most important variables in a model. Among the metabolites, 25 metabolites (xylose, pyroglutamic acid, fumaric acid, asparagine, phosphoric acid, glutamine, ferulic acid, quinic acid, raffinose, aspartic acid, cysteine, mannose, glycerol, glyceric acid, threonine, beta-alanine, inositol, glutamic acid, valine, tyrosine, isoleucine, oxalic acid, 4-aminobutyric acid, succinic acid, and glycolic acid) were identified, as these compounds had a significant VIP value (Figure S9). These findings indicate that there are significant differences in the primary metabolism of *L*. *radiata* flowers from stages 1 to 4.

**Figure 4:**
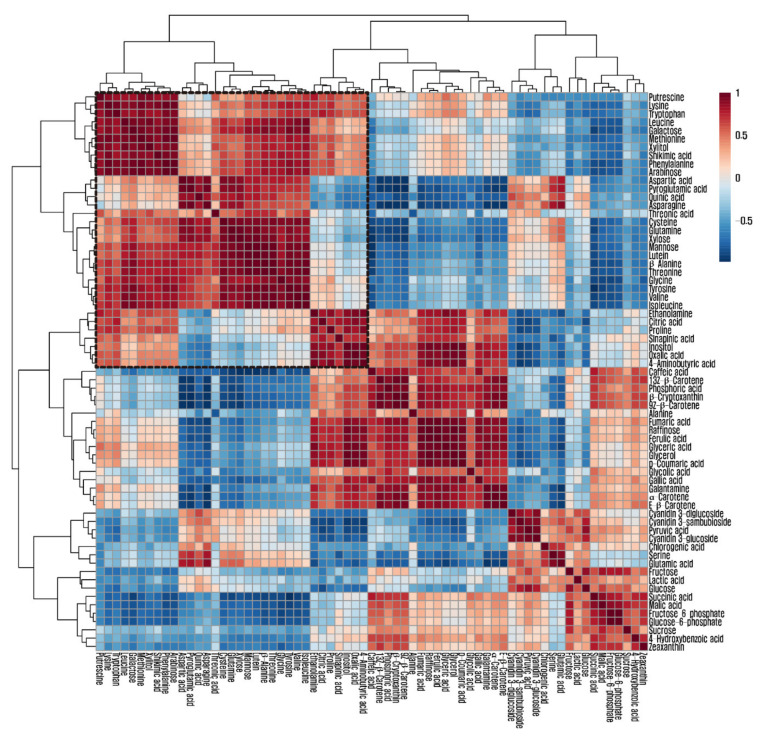
Correlation matrix and cluster analysis of results obtained from data on 64 metabolites for four flower developmental stages of *L*. *radiata*. Each square shows the Pearson’s correlation coefficient for a pair of compounds, and the value for the correlation coefficient is represented by the intensity of the blue or red color, as indicated on the color scale.

A hierarchical cluster analysis was carried out using the Pearson’s correlation results to obtain insights into the relationships between the 64 metabolites from stages 1 to 4 of the *L*. *radiata* flowers (Figure 4). Most amino acids were grouped together using the Pearson’s correlation and average linkage clustering methods. Glutamine, glutamic acid, aspartic acid, and asparagine comprise the metabolic network involved in plant nitrogen assimilation. Glutamic acid was positively correlated with aspartic acid (*r* = 0.78601, *p* = 0.0024) and asparagine (*r* = 0.90477, *p* < 0.0001), whereas glutamine was positively correlated with aspartic acid (*r* = 0.9129, *p* < 0.0001) and asparagine (*r* = 0.85446, *p =* 0.0004). The aspartate family of amino acids consists of lysine, beta-alanine, asparagine, methionine, threonine, and isoleucine. There were strong correlations between aspartic acid and the amino acids of the aspartate family, including isoleucine (*r* = 0.66757, *p* = 0.0177), beta-alanine (*r* = 0.73026, *p* = 0.007), asparagine (*r* = 0.94446, *p* < 0.0001), and threonine (*r* = 0.76929, *p* = 0.0034), as well as a strong correlation between aspartic acid and amino acids derived from 3-phosphoglycerate, including serine (*r* = 0.67589, *p* = 0.0158), glycine (*r* = 0.78311, *p* = 0.0026), and cysteine (*r* = 0.91615, *p* < 0.0001).

In this study, the pools of glutamine, glutamic acid, asparagine, and aspartic acid, which play important roles in nitrogen assimilation and metabolism into diverse amino acids, revealed an increasing pattern during flower development and led to increasing patterns of pyroglutamic acid, glycine, serine, methionine, leucine, valine, tyrosine, threonine, lysine, cysteine, isoleucine, and phenylalanine. In particular, this is supported by the strong positive correlations between glutamine, glutamic acid, asparagine, and aspartic acid and other amino acids, which showed increasing patterns. Decreasing patterns of fumaric acid, malic acid, and succinic acid from the TCA cycles supported the increasing patterns of glutamic acid, since both succinic acid and glutamic acid are derived from the same precursor, *α*-ketoglutarate. In addition, strong negative correlations between glutamic acid and TCA intermediates (citric acid, fumaric acid, malic acid, and succinic acid) were observed. Consistent with these findings, previous studies reported that the asparagine biosynthesis pathway is active in flowers. Le et al. [48] reported that the transcription of ASPARAGINE SYNTHETASE1 (*ASN1*), which encodes an enzyme that transfers amide N from glutamine to asparagine, increased during the life cycle of *Arabidopsis* flowers. Additionally, the expression level of *ASN1* was significantly higher than that of rosette, cauline, stem, and roots in wild-type *Arabidopsis*, and the asparagine content increased and then decreased during flower development together with *ASN1* expression levels [49]. Jia et al. [50] reported an increase in serine, alanine, valine, and threonine with a decrease in TCA cycle intermediates, such as malic acid and citric acid during flower development and senescence in *N*. *tazetta* ‘Kashmir Local’, which is a plant that belongs to the Liliaceae. A gradual increase in α-amino acid content was concomitant with our results, showing increasing patterns of α-amino acids including alanine, asparagine, aspartic acid, cysteine, glutamic acid, glutamine, glycine, isoleucine, leucine, lysine, methionine, phenylalanine, proline, serine, threonine, tryptophan, tyrosine, and valine [34]. Waseem and Inayatullah [35] reported an increase in α-amino acid content, while flowers of *C*. *ajacis* cv. Violet blue opened and senesced. Not only did α-amino acids gradually increase in the different flower development stages of *R*. *asiaticus* L. [36], but free amino acids also showed an increasing pattern in the different developmental stages of flower buds in *Spathodea campanulata* P. Beauv. [51].

## 4. Conclusions

In this study, a total of 18 secondary metabolites, including three anthocyanins, seven carotenoids, seven phenolic acids, and one alkaloid, as well as 49 primary metabolites, including an amine, sugars, sugar intermediates, sugar alcohols, amino acids, organic acids, and TCA cycle intermediates, were detected in the flowers of *L*. *radiata* at four developmental stages. Most secondary metabolites showed decreasing patterns in terms of flower development and senescence. Similarly, decreasing patterns of total sugars, consisting mainly of sugars, sugar alcohols, and sugar intermediates, were observed. These correlations might be due to the involvement of sugars as energy sources and precursors for metabolic processes. Flower colors were mainly imparted by the identified anthocyanins and carotenoids. In particular, the accumulation patterns of anthocyanins were correlated to intensity of petal colors during flower development and senescence. Among the primary metabolites, increasing patterns of amino acids were negatively correlated with the accumulation patterns of TCA cycle intermediates, which are biosynthetically linked to amino acid metabolism. Therefore, this study provides a systematic report on the composition of primary metabolites and secondary metabolites in the flowers of *L*. *raidata* at the four developmental stages.

## Figures and Tables

**Table 1:** HPLC analysis of carotenoids in the different flowering stages of *L*. *radiata*.

Compound (µg/g)	Stage 1	Stage 2	Stage 3	Stage 4
Lutein	16.12 ± 1.50c ^1^	18.05 ± 1.71c	22.37 ± 3.30b	47.99 ± 1.00a
Zeaxanthin	7.63 ± 0.26ab	8.15 ± 0.44a	7.59 ± 0.26ab	7.19 ± 0.07b
β-Cryptoxanthin	17.43 ± 0.58a	16.58 ± 0.44a	10.93 ± 1.15b	9.27 ± 0.22c
13*Z*-β-Carotene	20.69 ± 1.25a	22.25 ± 0.54a	11.43 ± 2.25b	10.22 ± 0.49b
α-Carotene	2.37 ± 0.18a	1.97 ± 0.30b	0.93 ± 0.17c	1.04 ± 0.05c
β-Carotene	89.15 ± 4.85a	80.75 ± 2.08a	42.10 ± 7.36b	50.31 ± 0.70b
9*Z*-β-Carotene	77.60 ± 4.15a	68.87 ± 2.31b	28.81 ± 6.26c	12.26 ± 0.80d

^1^ Mean values marked with different letters were significantly different (*p* < 0.05, ANOVA, DMRT).

**Table 2:** HPLC analysis of galantamine in the different flowering stages of *L*. *radiata*.

Flower Developmental Stages	Galantamine (mg/g DW)
Stage 1	2.71 ± 0.13a ^1^
Stage 2	2.39 ± 0.17b
Stage 3	1.65 ± 0.01d
Stage 4	1.89 ± 0.08c

^1^ Mean values with different letters were significantly different (*p* < 0.05, ANOVA, DMRT).

**Table 3:** HPLC and GC-TOFMS analysis of phenolic acids in the different flowering stages of *L*. *radiata*.

Equipment	Compound	Stage 1	Stage 2	Stage 3	Stage 4
HPLC (µg/g)	Gallic acid	74.51 ± 5.50a ^1^	36.99 ± 4.55b	34.63 ± 6.74b	13.38 ± 0.46b
	4-Hydroxybenzoic acid	60.08 ± 4.64ab	65.40 ± 26.23a	51.82 ± 34.68ab	17.86 ± 11.92b
	Chlorogenic acid	83.95 ± 15.74b	108.48 ± 27.79ab	222.52 ± 121.59a	125.98 ± 12.83ab
	Caffeic acid	143.96 ± 17.31a	110.98 ± 6.95b	104.00 ± 5.94b	24.66 ± 0.00c
	*p*-Coumaric acid	26.24 ± 2.36a	4.12 ± 2.82b	1.10 ± 0.81bc	N.D
GC-TOFMS (Intensity/g)	Ferulic acid	0.06 ± 0.01a	0.04 ± 0.00b	0.02 ± 0.00c	0.02 ± 0.00c
	Sinapinic acid	0.08 ± 0.01a	0.07 ± 0.01ab	0.05 ± 0.00c	0.06 ± 0.01bc

^1^ Mean values marked with different letters were significantly different (*p* < 0.05, ANOVA, DMRT).

**Table 4:** HPLC analysis of anthocyanin in the different flowering stages of *L*. *radiata*.

Compound (mg/g)	Stage 1	Stage 2	Stage 3	Stage 4
Cyanidin 3-diglucoside	0.04 ± 0.03b ^1^	0.14 ± 0.04a	0.16 ± 0.06a	0.12 ± 0.04ab
Cyanidin 3-sambubioside	3.94 ± 2.04c	11.69 ± 0.22ab	12.25 ± 0.26a	9.27 ±1.55b
Cyanidin 3-glucoside	0.04 ± 0.02c	0.17 ± 0.04a	0.15 ± 0.03ab	0.11 ± 0.01b

^1^ Mean values marked with different letters were significantly different (*p* < 0.05, ANOVA, DMRT).

## Supplementary Materials

The following are available online at https://www.mdpi.com/2218-273X/11/2/248/s1. Figure S1: HPLC carotenoid chromatogram obtained from flower of *Lycoris radiata.* Figure S2: HPLC galantamine chromatogram obtained from flower of *Lycoris radiata*. Figure S3: Expression of phenylpropanoid and galantamine biosynthesis genes in the different flowering stages of *L*. *radiata*. Figure S4: HPLC phenolic acid chromatogram obtained from flower of *Lycoris radiate*. Figure S5: HPLC anthocyanin chromatogram obtained from flower of *Lycoris radiata*. Figure S6: Representative chromatogram of metabolites obtained from L. radiata flower. Figure S7: Heatmap representing differences in relative metabolite concentration changes in the different developmental stages of L. radiata flowers. Increasing and decreasing contents of metabolites are shown in red and blue, respectively. Figure S8. (a) Scores and (b) loading plots of the principal component analysis (PCA) model obtained from 51 metabolites from *L*. *radiata* at the different flower developmental stages using GC-TOFMS. Figure S9: Variable importance in the projection (VIP) values of 51 metabolites derived from the partial least-squares discriminant analyses (PLS-DA) model of *L*. *radiata* at different flower developmental stages using GC-TOFMS.
