# Supplementary material for: Profiles of Secondary Metabolites (Phenolic Acids, Carotenoids, Anthocyanins, and Galantamine) and Primary Metabolites (Carbohydrates, Amino Acids, and Organic Acids) during Flower Development in Lycoris radiata
Source: Biomolecules. 2021 Feb 9;11(2):248. doi: 10.3390/biom11020248 (PMC7914633; doi:10.3390/biom11020248)
Supplement: Supplementary file 1 [file biomolecules-11-00248-s001.pdf]

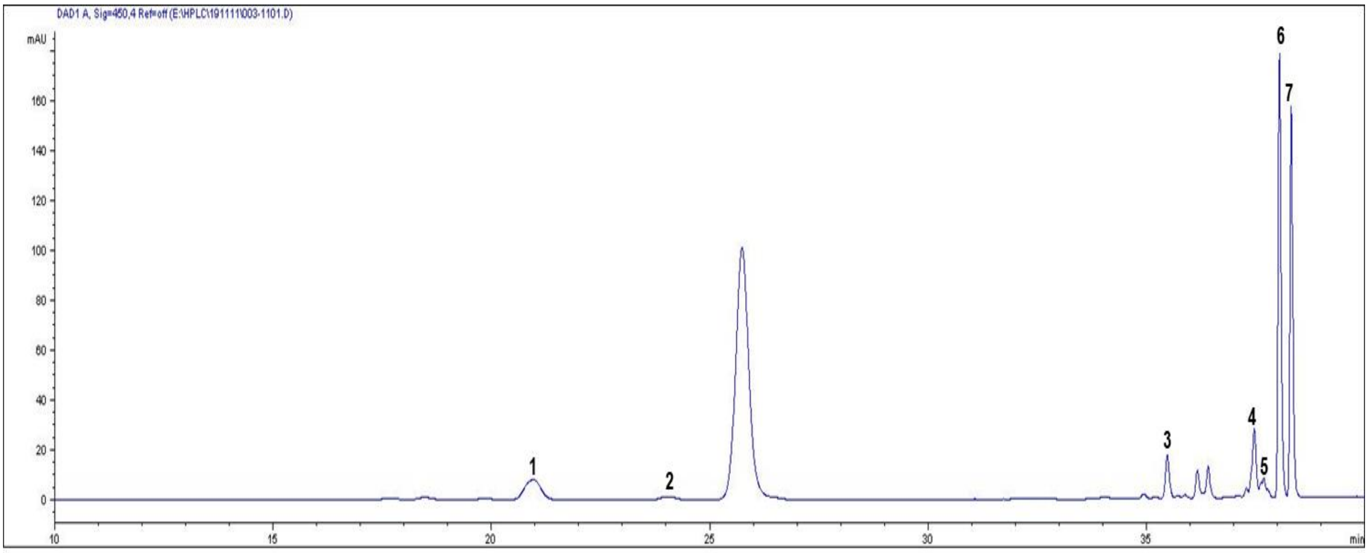

Figure S1. HPLC carotenoid chromatogram obtained from flower of *Lycoris radiata.* Peak: 1. Lutein; 2. Zeaxanthin; 3. β-Cryptoxanthin; 4. 13Z-β-Carotene; 5. α-Carotene; 6. β-Carotene; 7. 9Z-β-Carotene.

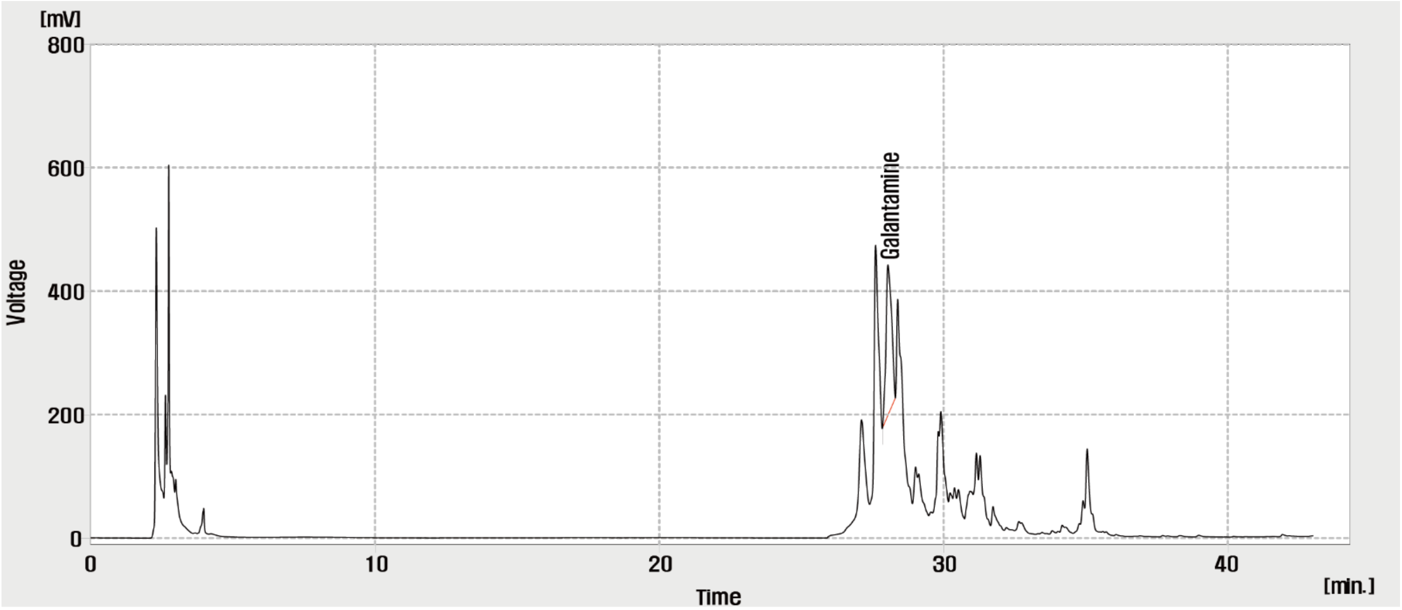

Figure S2. HPLC galantamine chromatogram obtained from flower of *Lycoris radiata*.

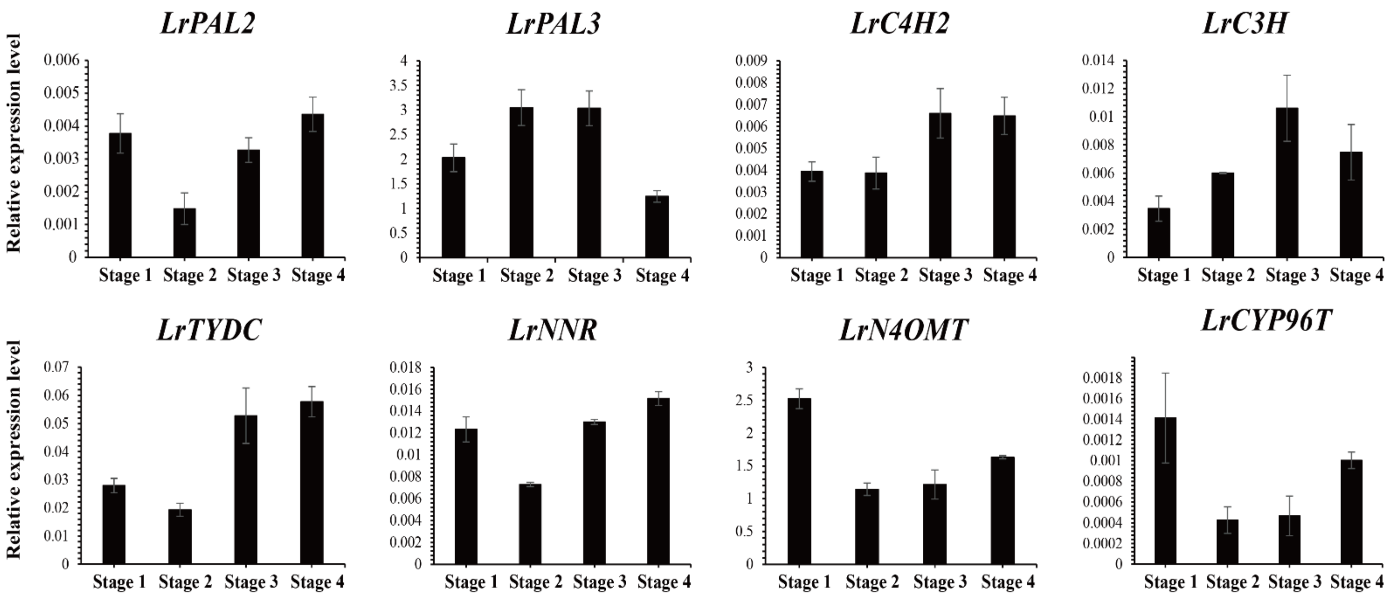

Figure S3. Expression of phenylpropanoid and galantamine biosynthesis genes in the different flowering stages of *L. radiata.*

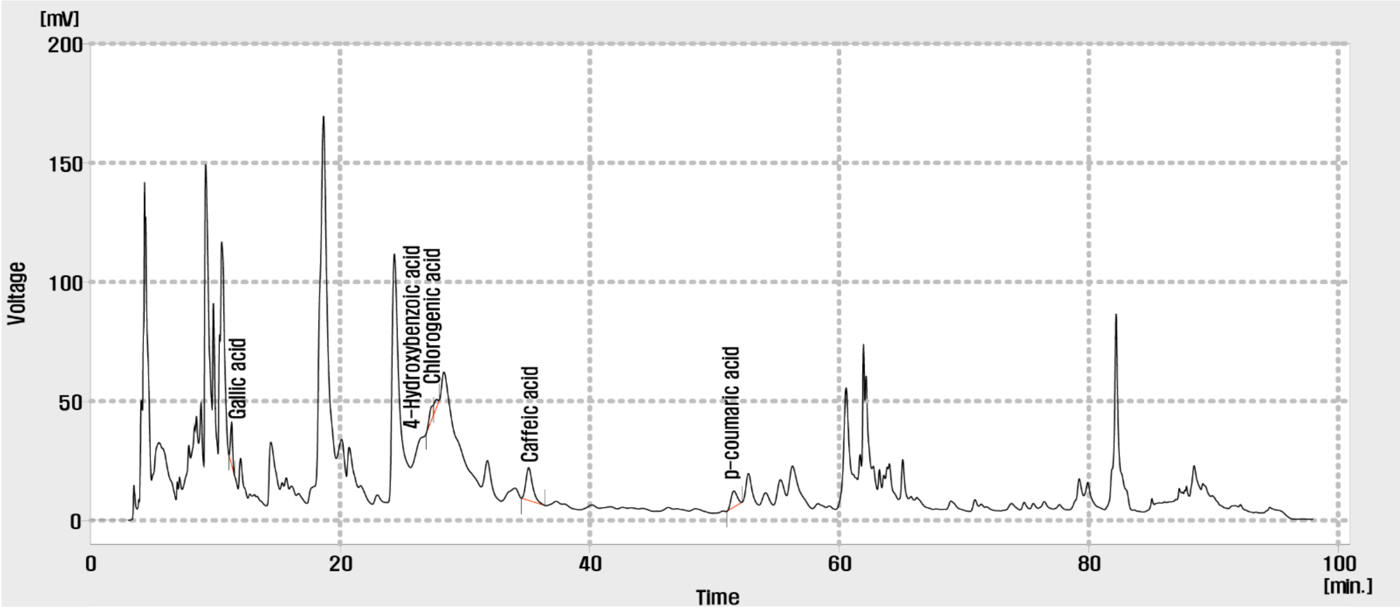

Figure S4. HPLC phenolic acid chromatogram obtained from flower of *Lycoris radiata.*

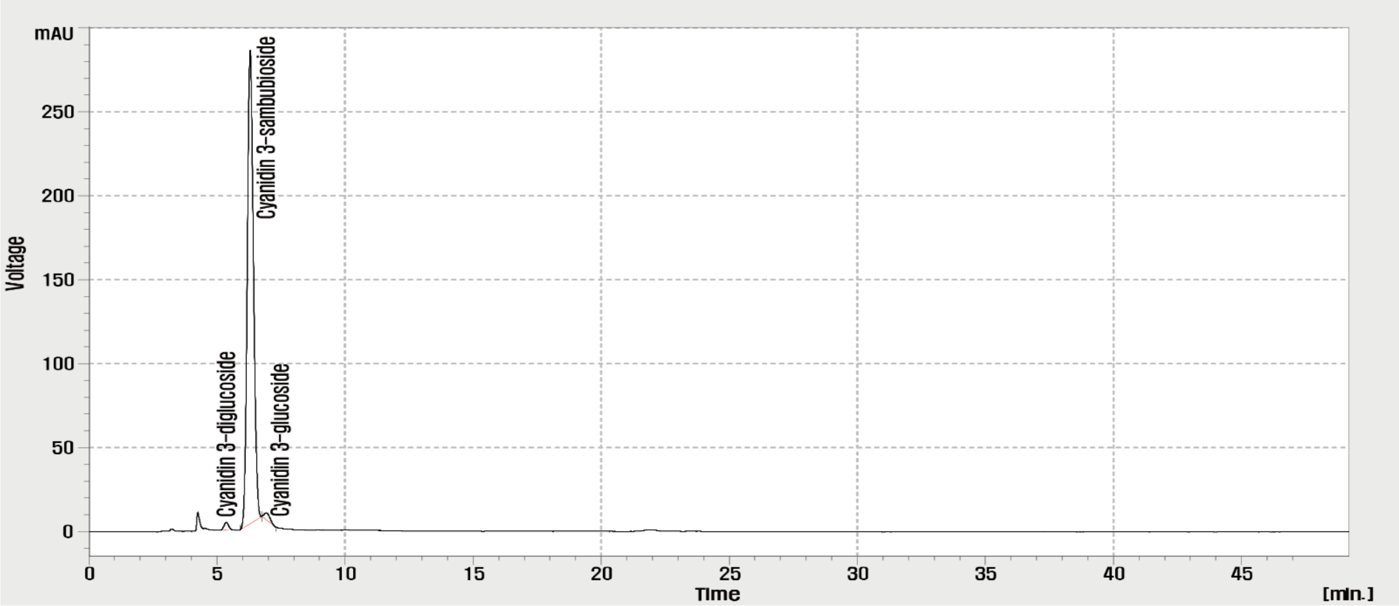

Figure S5. HPLC anthocyanin chromatogram obtained from flower of *Lycoris radiata.*

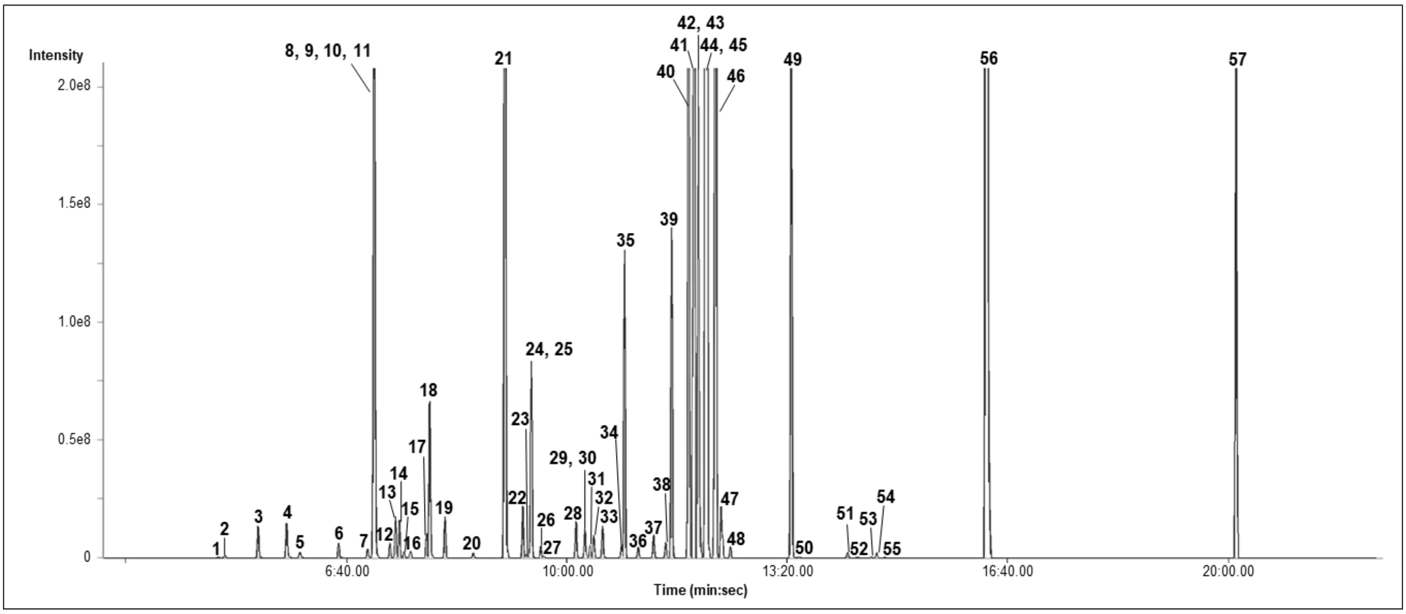

Figure S6. Representative chromatogram of metabolites obtained from L radiata flower. Peak: 1, Pyruvic acid; 2, Lactic Acid; 3, Alanine; 4, Oxalic acid; 5, Glycolic acid; 6, Valine; 7, Serine 1; 8, Ethanolamine; 9, Phosphoric acid; 10, Glycerol; 11, Leu cine; 12, Isoleucine; 13, Proline; 14, Glycine; 15, Succinic acid; 16, Glyceric acid; 17, Fumaric acid; 18, Serine-2; 19, Threonine; 20, β-Alanine; 21, Malic acid; 22, Aspartic acid; 23, Methionine; 24, Pyroglutamic acid; 25, 4-Aminobutyric acid; 26, Threonic acid; 27, Cysteine; 28, Glutamic acid; 29, Phenylalanine; 30, Xylose-1; 31, Xylose-2; 32, Arabinose; 33, Asparagine; 34, Xylitol; 35,Ribitol (internal standard); 36, Putrescine; 37, Glutamine; 38, Shikimic acid; 39, Citric acid; 40, Quinic acid; 41, Fructose-1; 42, Fructose-2; 43, Mannose; 44, Galactose; 45, Glucose-1; 46, Glucose-2; 47, Lysine; 48, Tyrosine; 49, Inositol; 50, Ferulic acid; 51, Tryptophan; 52, Sinapinic acid; 53, Fructose-6-phosphate; 54, Glucose-6-phosphate-1; 55, Glucose-6-phosphate-2; 56, Sucrose; 57, Raffinose.

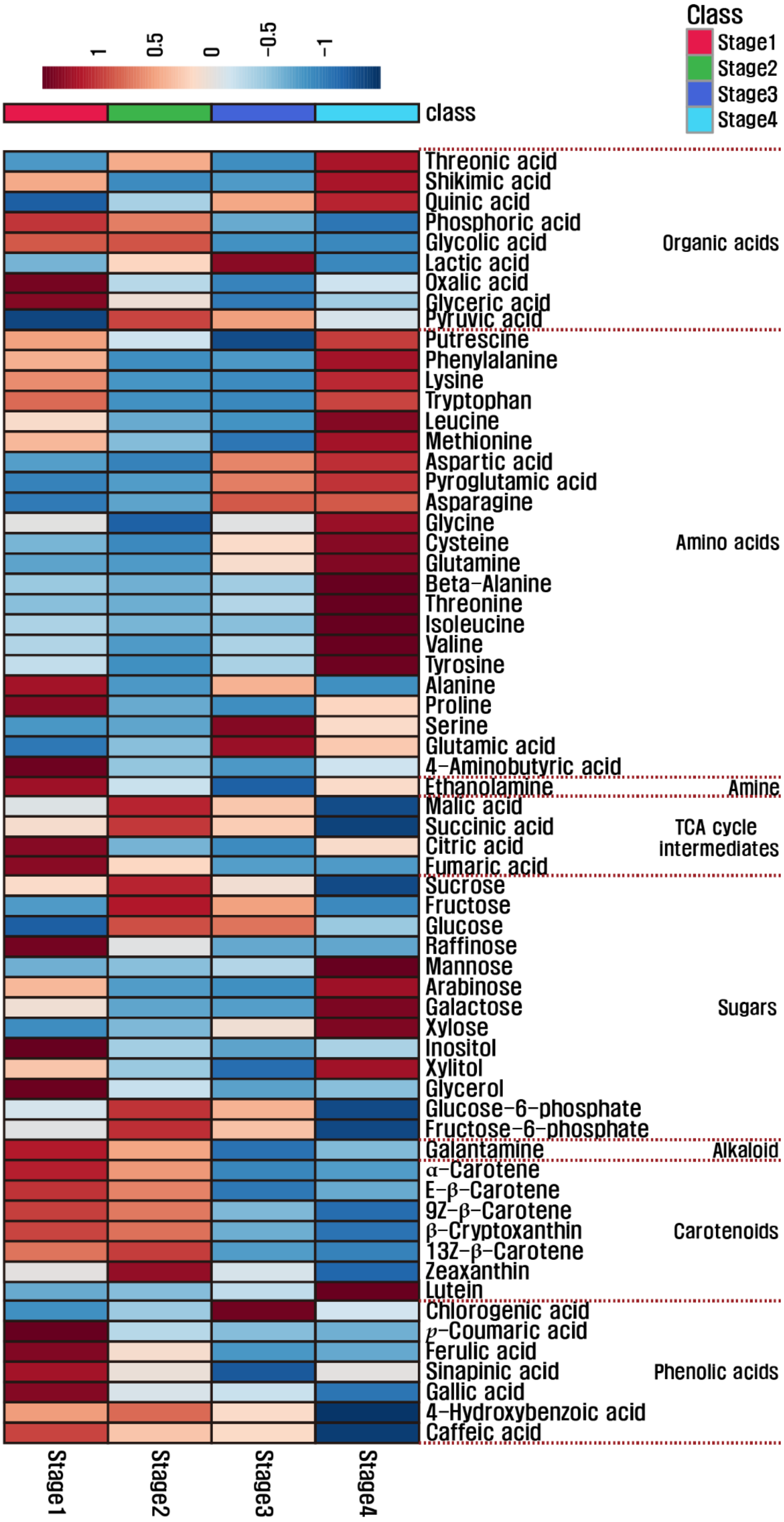

Figure S7. Heatmap representing differences in relative metabolite concentration changes in the different developmental stages of *L. radiata* flowers. Increasing and decreasing contents of metabolites are shown in red and blue, respectively.

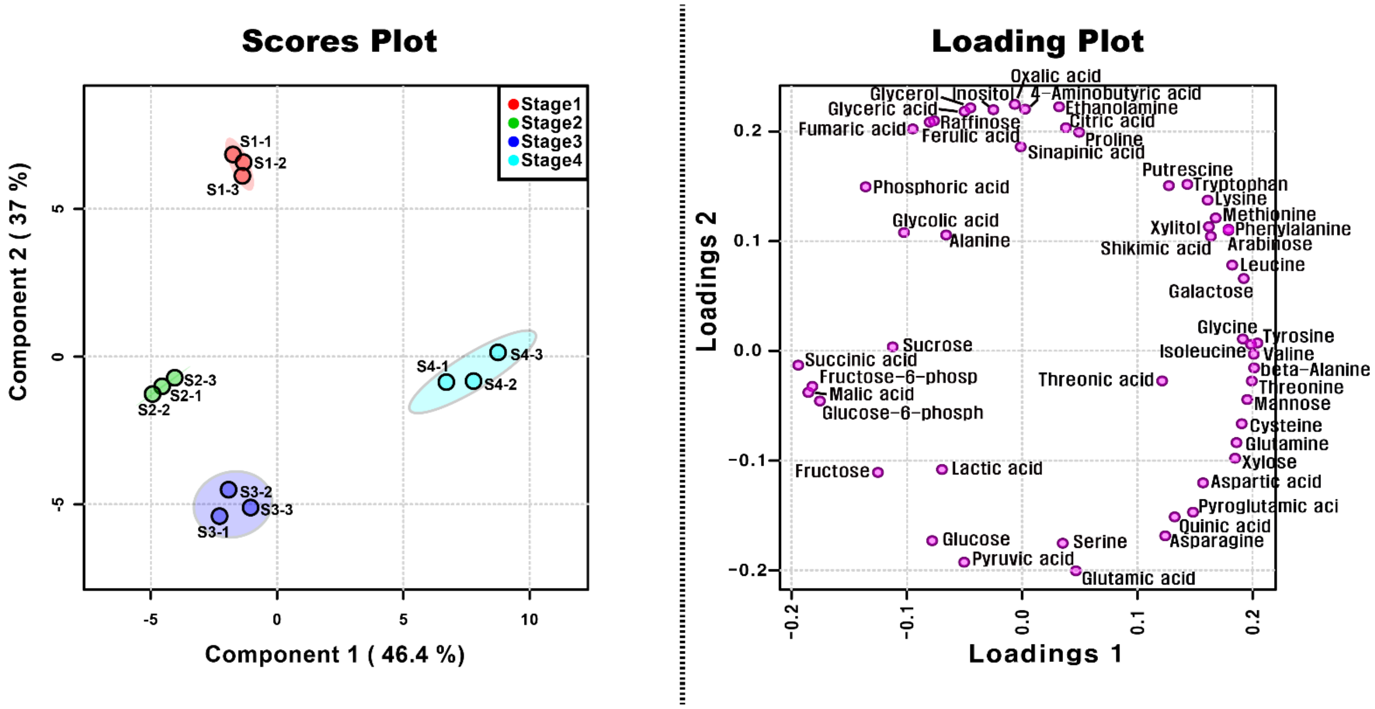

Figure S8. (**A**) Scores and (**B**) loading plots of the principal component analysis (PCA) model obtained from 51 metabolites from *L. radiata*at the different flower developmental stages using GC-TOFMS.

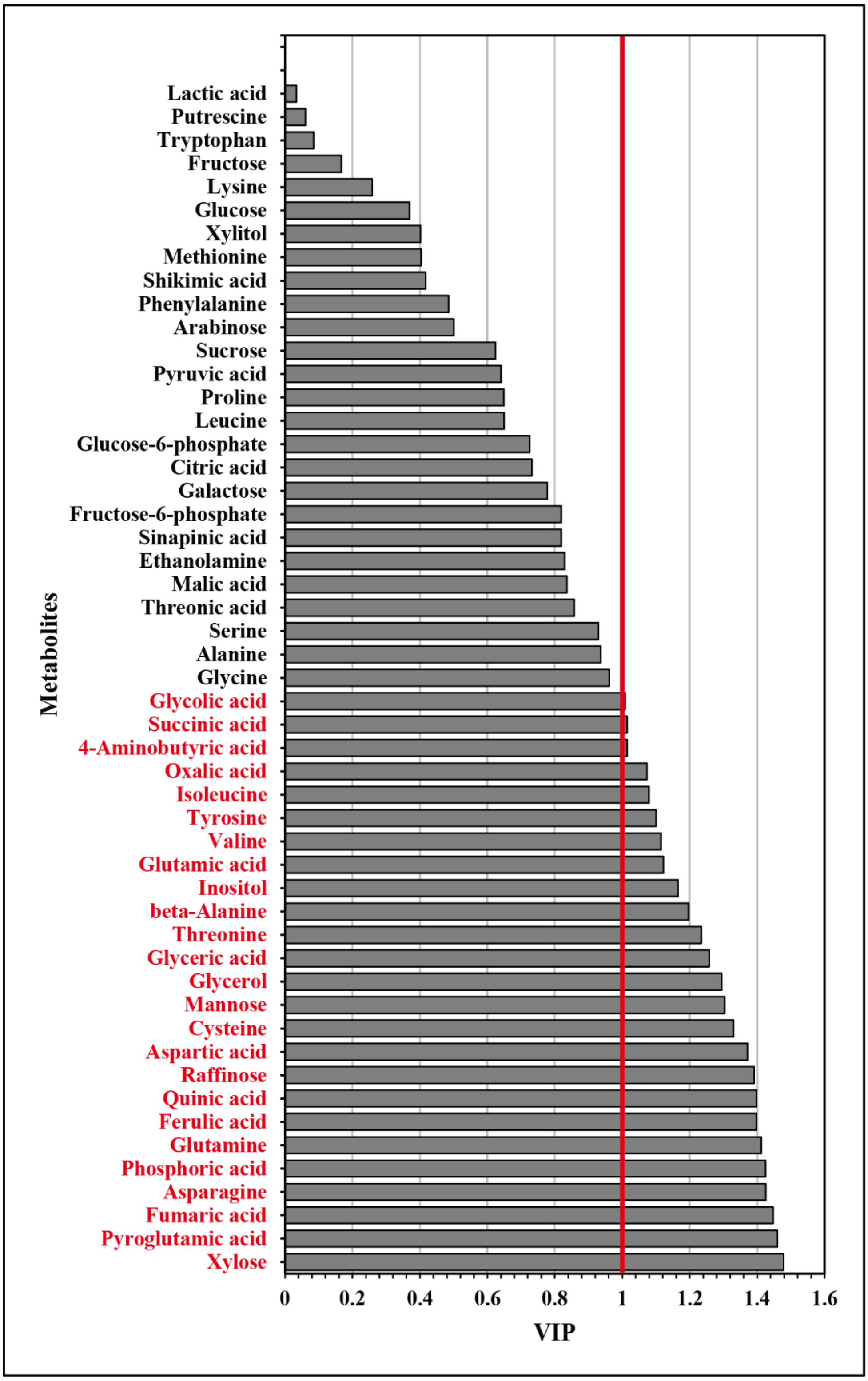

Figure S9. Variable importance in the projection (VIP) values of 51 metabolites derived from the partial least-squares discriminant analyses (PLS-DA) model of *L. radiata*at different flower developmental stages using GC-TOFMS.
